# Autoimmune Myelitis and Myocarditis in a Patient With Anti-Aquaporin-4, Antinuclear, and Antiphospholipid Autoantibodies: The Neuromyelitis Optica-Systemic Lupus Erythematosus (NMO-SLE) Overlap Syndrome

**DOI:** 10.7759/cureus.31334

**Published:** 2022-11-10

**Authors:** Edward C Mader, Olinda Verdecie, Vaniolky Losada, Jesus F Lovera

**Affiliations:** 1 Neurology, Louisiana State University (LSU) Health Sciences Center, New Orleans, USA

**Keywords:** neuromyelitis optica, nmo, lupus, sle, transverse myelitis, letm, myocarditis, multiple autoimmune syndrome, antibodies, aquaporin-4

## Abstract

The coexistence of two or more autoimmune diseases is well-known, e.g., a person can have neuromyelitis optica (NMO) and systemic lupus erythematosus (SLE) at the same time. We report a case of NMO-SLE overlap syndrome with myelitis and myocarditis as the initial manifestations. The patient, a 64-year-old man, presented with a 15-day history of ascending sensory loss and a 10-day history of exertional dyspnea. Magnetic resonance imaging (MRI) revealed longitudinally extensive transverse myelitis (LETM) from C7 to T6. Serology showed a high anti-aquaporin-4 antibody level. We diagnosed NMO based on these findings. Echocardiography showed a hypokinetic left ventricle with a severely reduced ejection fraction. Cardiac MRI demonstrated delayed gadolinium enhancement in the myocardium consistent with active inflammation. Because the cardiac findings could not be explained on the basis of NMO, we started searching for another autoimmune disease. Serology came back positive for a variety of autoantibodies, including antinuclear, anti-dsDNA, anti-chromatin, anti-cardiolipin, anti-β2-glycoprotein-1, and lupus anticoagulant. These findings, along with leukopenia and low serum complement C4, prompted us to diagnose SLE, in addition to NMO. He was initially treated with plasmapheresis and methylprednisolone. Maintenance therapy consisted of rituximab, hydroxychloroquine, and aspirin. One year later, he only complained of mild paresthesia in the feet. Patients with NMO should always be screened for SLE especially if they have signs and symptoms that cannot be accounted for by NMO alone, e.g., our patient had myocarditis. Conversely, patients with SLE and evidence of transverse myelitis should be screened for anti-AQP4 antibodies.

## Introduction

Neuromyelitis optica (NMO) is an autoimmune disease of the central nervous system (CNS) with aquaporin-4 (AQP4) channels as the primary targets of the autoimmune process [1]. AQP4 channels are ubiquitous but they are particularly abundant in astrocytes [2]. NMO spectrum disorder (NMOSD) includes anti-AQP4 immunoglobulin G (IgG) positive NMO with typical or atypical MRI lesions, anti-AQP4 IgG-negative NMO, and NMO with unknown anti-AQP4 IgG status [3,4]. The International Panel for NMO Diagnosis (IPND) identified six NMO core clinical characteristics: 1) optic neuritis, 2) acute myelitis, 3) area postrema syndrome, 4) acute brainstem syndrome, 5) narcolepsy or acute diencephalic syndrome with typical NMOSD diencephalic lesions, and 6) cerebral syndrome with typical NMOSD brain lesions [4]. The 2015 IPND diagnostic criteria for NMO in adults require at least one core clinical characteristic if anti-AQP4 IgG is positive [4]. If anti-AQP4 IgG is negative or indeterminate, at least two core clinical characteristics must be present, with at least one being optic neuritis, longitudinally extensive (≥3 vertebral segments) transverse myelitis (LETM), or area postrema syndrome [4].

Systemic lupus erythematosus (SLE) is a multi-organ autoimmune disease characterized by a wide array of autoantibodies, immune complex deposition, and inflammation of various organs or tissues, such as the joints, skin, kidneys, serosal membranes, and the CNS [5]. The European League Against Rheumatism (EULAR) and the American College of Rheumatology (ACR) updated the criteria for classifying SLE in 2021 [6]. Antinuclear antibody (ANA) titer ≥1:80 was defined as the entry criteria. The clinical criteria were organized into six domains (constitutional, hematologic, neuropsychiatric, mucocutaneous, serosal, musculoskeletal, and renal) and the immunological criteria into three domains (antiphospholipid antibodies, complement proteins, and SLE-specific antibodies) [6]. Each criterion under each of the clinical and immunological domains was assigned a weight, e.g., under the hematologic domain, leukopenia was assigned a weight of 3, thrombocytopenia a weight of 4, and autoimmune hemolysis a weight of 4 [6]. According to the 2021 EULAR-ACR criteria, a disease can be classified as SLE if: (1) the ANA titer is ≥1:80, (2) at least one clinical criteria is present, and (3) the sum of the highest weights of each clinical and immunological domain is ≥10 [6].

NMO may be comorbid with another systemic or organ-specific autoimmune disorder [7]. The most commonly diagnosed systemic autoimmune diseases in patients with NMO are Sjogren syndrome (SS) and SLE [8,9]. The simultaneous presence of NMO and SS/SLE should be viewed as a “coexistence” rather than as a “complication” [6]. There is no predictable sequence of disease occurrence in patients with NMO-SLE overlap syndrome: cases have been reported in which NMO preceded SLE, NMO followed SLE, and NMO and SLE occurred at the same time [9]. We report a case of NMO-SLE overlap syndrome with clinical manifestations and MRI evidence of LETM and myocarditis and positive serology for anti-AQP4, anti-nuclear, and antiphospholipid autoantibodies.

## Case presentation

A 64-year-old man with type 2 diabetes, hypertension, and hypothyroidism presented with ascending numbness, gait ataxia, and exertional dyspnea. He already had numbness in the legs and feet for two months prior to admission, most likely due to lumbar stenosis (see MRI results below). Fifteen days prior to admission, the numbness started to spread, first to his thighs, then his abdomen, and finally his chest up to the T5 dermatome. He also noted increasing difficulty with ambulation, especially in poorly lit places. Ten days prior to admission, he started having dyspnea on exertion, which progressively worsened motivating him to go to the emergency room. At home, he took metformin, hydrochlorothiazide, enalapril, and levothyroxine, on examination, he was alert and fully oriented with no complaint of dyspnea at rest. Blood pressure was 152/84 mmHg, pulse rate 58/min, respiratory rate 24/min, temperature 36.3 ºC, and blood oxygen saturation 98%. Brudzinski and Kernig signs were absent. The only abnormal findings on the neurological exam were reduced touch sensation below the T5 dermatome, absent position and reduced vibration sense in both feet and ankles, absent Achilles reflex in both ankles (other reflexes were 1+), and positive Romberg sign.

**Figure 1:**
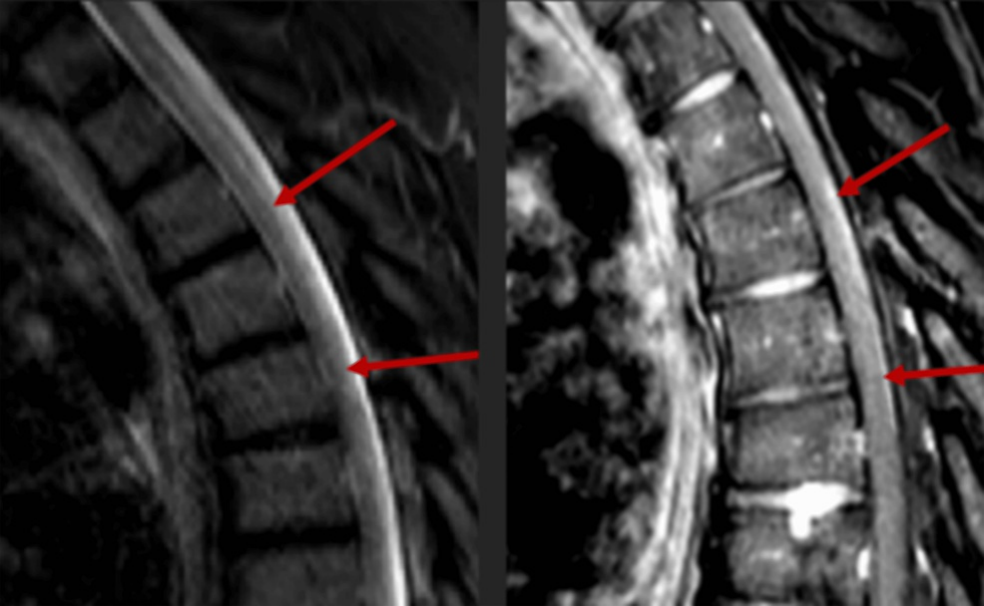
MRI of the spine showing a hyperintense signal on short tau inversion recovery (STIR) sequence (left) and gadolinium enhancement on T1-weighted image (right) indicating active inflammation in the posterior cord extending from the C7 to the T5 vertebrae (arrows). These findings are consistent with longitudinally extensive transverse myelitis.

**Figure 2:**
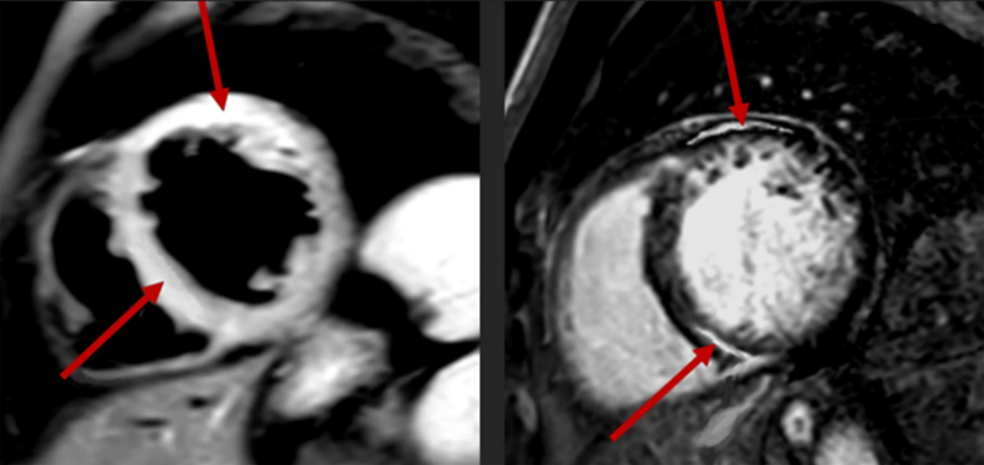
MRI of the heart showing hyperintense signal on the T2-weighted image (left) and delayed gadolinium enhancement on the T1-weighted image (right) indicating active inflammation in the anterior and inferoseptal walls of the left ventricle (arrows). The right ventricle myocardium was also involved but to a much lesser extent (not shown). These findings are consistent with acute autoimmune myocarditis.

Chest x-ray showed pulmonary vascular congestion. Electrocardiography showed transient episodes of supraventricular tachycardia. Magnetic resonance imaging (MRI) of the spine showed LETM extending from the C7 to the T5 vertebrae (Figure 1). Bilateral L4-L5 and L5-S1 neuroforaminal stenoses were also noted explaining his initial sensory symptoms as described above. Echocardiography showed hypokinesis of the left ventricle with ejection fraction reduced to 20%. Left heart catheterization and coronary angiography were normal. Cardiac MRI showed signs of active inflammation in the left ventricle and, to a lesser extent, the right ventricle myocardium (Figure 2). Technetium pyrophosphate nuclear scintigraphy excluded amyloidosis and ischemia as the cause of cardiomyopathy.

Blood chemistry showed normal serum glucose, glycated hemoglobin (5.1%), electrolytes, ammonia, BUN, creatinine, transaminases, protein, albumin, IgG, thyroid stimulating hormone, and troponin 1 levels. Hematology test results (Table 1) showed leukopenia, mild anemia, and prolongation of the activated partial thromboplastin time (aPTT). The differential count, red cell indices, platelet count, prothrombin time, and erythrocyte sedimentation rate were all normal. Cerebrospinal fluid (CSF) test results (Table 2) showed high protein and normal glucose, white blood cells, red blood cells, and angiotensin-converting enzyme; myelin basic protein was present in the CSF but there were no oligoclonal bands. CSF albumin, lgG, lgG/albumin ratio, lgG index, and lgG synthesis rate were all within normal limits. Serology test results (Table 3) showed high levels of anti-AQP4, antinuclear, anti-dsDNA, anti-chromatin, anti-cardiolipin, and anti-β2-glycoprotein-1 antibodies. Serum was also positive for lupus anticoagulant, which was confirmed by prolongation of the aPTT, the dilute Russel viper venom time, and the silica clot time. Serum complement C4 was low while complement C3 was normal. Interleukin-2 soluble receptor levels were within normal limits and anti-myelin oligodendrocyte glycoprotein antibodies were not detected.

**Table 1:** Hematology test results *Retested five months after discharge, INR: international normalized ratio for prothrombin time, aPTT: activated partial thromboplastin time, ESR: erythrocyte sedimentation rate

	Admission	Reference Range	Follow-up*
White blood cells	2.5 x 10^3^/µL	4.5-11.0 x 10^3^/µL	9.0 x 10^3^/µL
Red blood cells	4.29 x 10^6^/µL	4.50-5.90 x 10^6^/µL	3.32 x 10^6^/µL
Hemoglobin	12.5 g/dL	13.5-17.5 g/dL	9.2 g/dL
Hematocrit	37.4 %	40.0-51.0 %	29.6 %
Platelets	214 x 10^3^/µL	130-400 x 10^3^/µL	196 x 10^3^/µL
Prothrombin time	12.7 seconds	10.0-13.0 seconds	11.7 seconds
INR	1.1	0.9-1.1	1.0
aPTT	38.2 seconds	24.0-37.0 seconds	32.8 seconds
ESR	13 mm/h	≤20 mm/h	6 mm/h

**Table 2:** Cerebrospinal fluid test results ACE: angiotensin-converting enzyme, IgG: immunoglobulin G

	Admission	Reference Range
Protein	80.8 mg/dL	15.0-45.0 mg/dL
Glucose	60 mg/dL	40-70 mg/dL
White blood cells	2 /µL	0-5 /µL
Red blood cells	5 /µL	0-5 /µL
ACE	2.3 U/L	0.0-2.5 U/L
Albumin	44 mg/dL	11-48 mg/dL
lgG	6.0 mg/dL	0.0-8.6 mg/dL
lgG/albumin ratio	0.14	0.00-0.25
lgG index	0.4	0.0-0.7
lgG synthesis rate	-8.3 mg/day	-9.9-+3.3 mg/day
Myelin basic protein	2.6 ng/mL	0.0-1.2 ng/mL
Oligoclonal bands	0	0-3

**Table 3:** Serology test results *Retested five months after discharge, **except for anti-AQP4 and anti-dsDNA antibodies, which were retested a year after discharge, IgG: immunoglobulin G, AQP4: aquaporin-4, dsDNA: double-stranded DNA, MOG: myelin oligodendrocyte glycoprotein

	Admission	Reference	Follow-up*
Anti-AQP4 IgG	>80 U/mL	≤2.9 U/ml	negative^**^
Antinuclear antibodies	positive (≥1:640)	<1:80	N/A
Anti-dsDNA antibodies	positive (1:20)	negative	negative^**^
Anti-cardiolipin lgG	81.3 U/mL	<20.0 U/mL	66.5 U/mL
Anti-β2-glycoprotein-1 IgG	80.1 U/mL	<20.0 U/mL	79.3 U/mL
Lupus anticoagulant	positive	negative	positive
Dilute Russel viper venom	42.3 sec	25.5-13.3 sec	29.7 sec
Silica clot time	67.8 sec	26.2-25.2 sec	45.4 sec
Anti-chromatin antibodies	5.4 AI	<1.0 AI	N/A
Complement C3	112 mg/dL	83-180 mg/dL	101 mg/dL
Complement C4	12 mg/dL	18-55 mg/dL	13 mg/dL
Interleukin-2 receptor	9 pg/ml	≤1033 pg/ml	N/A
Anti-MOG antibodies	negative (<1:20)	negative (<1:20)	N/A

Acute immunotherapy consisted of five courses of plasmapheresis administered over a 10-day period and methylprednisolone 1,000-mg IV q24h for five days. Long-term immunotherapy consisted of rituximab 1,000-mg IV every six months and hydroxychloroquine 400-mg PO daily. He was also put on aspirin 81-mg PO once daily. Two weeks after discharge, he only complained of paresthesias in his legs and decreased vibration sense was the only abnormal examination finding. His left ventricular ejection fraction increased to >55%. One year later, he had a normal neurological examination and he only complained of occasional paresthesia in his feet, perhaps due to lumbar stenosis.

## Discussion

The NMO-SLE overlap syndrome is not common, but it is not rare either, with more than two dozen cases being reported in the literature [7-9]. What makes this case of NMO-SLE overlap syndrome unique is that both the spinal cord and the cardiac muscles were simultaneously involved in the autoimmune process. We used the 2015 IPND criteria to diagnose NMO [4] and the 2021 EULAR-ACR criteria to diagnose SLE [6] in our patient. The markedly elevated anti-AQP4 IgG level (>80 U/mL) and the presence of LETM on MRI were sufficient to diagnose NMO [4]. SLE was diagnosed on the basis of the high antinuclear antibody titer (1:640), the presence of one clinical criterion (leukopenia with a white cell count of 2.5 x 10^3^/µL), and the fact that we obtained a score of 14 when we added up the highest weight from each domain (three for leukopenia, two for anti-cardiolipin lgG or β2-glycoprotein-1 IgG or lupus anticoagulant, six for anti-dsDNA antibodies, and three for low C3 complement level) [6]. The revised 2018 Lake Louise criteria for acute myocarditis [10] were also fulfilled in our patient (a regional increase of signal intensity on T2-weighted MRI and regional late gadolinium enhancement on T1-weighted MRI in the anterior and inferoseptal walls of the left ventricle).

Transverse myelitis is strongly suggestive of NMO, especially when the MRI shows LETM [11]. However, LETM is not pathognomonic of NMO since it has been observed in a variety of infectious, neoplastic, and metabolic disorders, and many other autoimmune diseases, such as SLE [12,13]. Acute transverse myelitis may also be the initial manifestation of SLE [14,15]. Most CNS manifestations of SLE occur during high disease activity but transverse myelitis may occur during low or absent disease activity [14]. Many SLE patients with transverse myelitis test positive for antiphospholipid antibodies implying that thrombosis, in addition to vasculitis, has a role in the pathogenesis of lupus myelitis [16]. Both NMO and SLE myelitis have been associated with unfavorable outcome [14, 15]. Until we find a reliable way to distinguish SLE myelitis and NMO myelitis, we can only assume that NMO, rather than SLE, is the reason for LETM in patients with NMO-SLE overlap syndrome [11].

Autoimmune myocarditis as a feature of NMO is practically unknown: only one case of NMO with myocarditis has been hitherto reported [17]. The human heart expresses AQP4 at the protein level but the significance of this in relation to anti-AQP4 autoimmunity needs further investigation [18]. Because of the lack of experimental data linking NMO and cardiac disease, there is no reason to believe that NMO is the cause of myocarditis in our patient. Cardiac involvement in SLE may take the form of pericarditis, myocarditis, endocarditis, valvulitis, or coronary artery disease [19]. While only acute pericarditis has been included in the EULAR-ACR criteria for SLE (under the “serosal” domain) [6], SLE myocarditis is undoubtedly significant from a prognostic and therapeutic perspective [17].

It is poorly understood why two antibody-mediated autoimmune diseases, such as NMO and SLE, occur in the same patient. The coexistence between NMO and systemic autoimmune diseases may reflect a general susceptibility to antibody-mediated autoimmunity [20]. B cells have an important role in regulating many aspects of the immune response. Besides environmental factors, genetic abnormalities may result in B-cell dysregulation and give rise to multiple antibody-mediated autoimmune diseases [21].

## Conclusions

We are not aware of any report of NMO-SLE overlap syndrome where the patient presented simultaneously with acute transverse myelitis and acute myocarditis. Our patient fulfilled the 2015 IPND diagnostic criteria for NMO and the 2021 EULAR-ACR criteria for SLE. Patients with NMO should always be screened for SLE especially if they have signs and symptoms that cannot be accounted for by NMO alone, e.g., our patient had myocarditis. Conversely, patients with SLE and evidence of transverse myelitis should be tested for serum anti-AQP4 antibodies. Long-term treatment should target both NMO and SLE. Our patient’s transverse myelitis and myocarditis resolved completely with rituximab and hydroxychloroquine.
